# Diffusion of dilute gas in arrays of randomly distributed, vertically aligned, high-aspect-ratio cylinders

**DOI:** 10.3762/bjnano.8.7

**Published:** 2017-01-09

**Authors:** Wojciech Szmyt, Carlos Guerra, Ivo Utke

**Affiliations:** 1 Empa, Swiss Federal Laboratories for Materials Science and Technology, Laboratory for Mechanics of Materials and Nanostructures, Feuerwerkerstrasse 39, 3602 Thun, Switzerland; 2 AGH University of Science and Technology, Faculty of Physics and Applied Computer Science, al. A. Mickiewicza 30, 30-059 Krakow, Poland; 3 now working at: FHNW University of Applied Sciences and Arts Northwestern Switzerland, Institute of Polymer Engineering, Klosterzelgstrasse 2, 5210 Windisch, Switzerland

**Keywords:** dilute gas, gas transport, molecular diffusion, nanocylinders, random walk

## Abstract

In this work we modelled the diffusive transport of a dilute gas along arrays of randomly distributed, vertically aligned nanocylinders (nanotubes or nanowires) as opposed to gas diffusion in long pores, which is described by the well-known Knudsen theory. Analytical expressions for (i) the gas diffusion coefficient inside such arrays, (ii) the time between collisions of molecules with the nanocylinder walls (mean time of flight), (iii) the surface impingement rate, and (iv) the Knudsen number of such a system were rigidly derived based on a random-walk model of a molecule that undergoes memoryless, diffusive reflections from nanocylinder walls assuming the molecular regime of gas transport. It can be specifically shown that the gas diffusion coefficient inside such arrays is inversely proportional to the areal density of cylinders and their mean diameter. An example calculation of a diffusion coefficient is delivered for a system of titanium isopropoxide molecules diffusing between vertically aligned carbon nanotubes. Our findings are important for the correct modelling and optimisation of gas-based deposition techniques, such as atomic layer deposition or chemical vapour deposition, frequently used for surface functionalisation of high-aspect-ratio nanocylinder arrays in solar cells and energy storage applications. Furthermore, gas sensing devices with high-aspect-ratio nanocylinder arrays and the growth of vertically aligned carbon nanotubes need the fundamental understanding and precise modelling of gas transport to optimise such processes.

## Introduction

Arrays of vertically aligned nanowires and nanotubes with high-aspect ratio composed of various materials have been widely used in science and industry. Arrays of silicon nanowires [1–2] and carbon nanotubes [3] are undoubtedly the most popular high-aspect-ratio cylinder array systems. Moreover, there has been growing interest in nanocylinder systems composed of metals [4], and metal oxides [5–6] have become increasingly researched for their use as large-surface one-dimensional materials in a wide range of potential fields of applications such as photovoltaics [7], photocatalysis [8], organic electronics [9] and supercapacitors [10].

Surface functionalisation and nanoengineering of high-aspect-ratio nanocylinder arrays often involve the exposure of the structures to gas-phase chemicals, for instance, during the coating of nanotubes or nanowires with thin films employing techniques such as chemical vapour deposition (CVD) [11] or atomic layer deposition (ALD) [12–13]. Our recent study constitutes an example of the coating of vertically aligned carbon nanotubes (VACNTs) with monocrystalline anatase using ALD [14]. Arrays of nanocylinders are also used in gas sensing systems [15–16]. The increasing interest in surface functionalisation via gas phase techniques as well as gas sensing applications with high-aspect-ratio nanocylinder arrays has raised the need for the fundamental understanding and precise modelling of gas-transport specifics required in the optimisation of the aforementioned processes [17]. Furthermore, root growth of VACNTs with a CVD method has been reported to be limited by gas diffusion, which additionally emphasizes the need for a reliable method of gas-transport modelling [18–19]. The understanding of the gas-transport kinetics within nanocylinder arrays will be also crucial in the design and optimisation of the industrially up-scalable spatial ALD processes [20] carried out on such substrates. Although processes based on gas transport in various types of nanocylinder arrays have been extensively studied for years, rather less attention has been paid to modelling of the diffusion phenomena. Diffusion modelling was attempted in the context of ALD coatings of CNTs [17] or VACNT growth [19,21–22]. These studies deliver rough estimates of the gas transport and lack rigidly derived formulae of gas diffusion inside a nanocylinder array. Most studies have been so far devoted to gas transport within more classic porous structures such as narrow holes or trenches constituting a foundation of the considerations of this study. The theory of molecular diffusion across nanopores has been developed by Martin Knudsen more than a century ago [23–24]. Knudsen’s theory handles surface collisions of molecules by diffusive reflection (following Lambert’s cosine law) and neglects specular reflection. It has been successfully and widely tested for microchannels and nanopores [25–26], as well as for ALD growth in nanopores [27–31], and conformal CVD [32] considering kinetic parameters of surface sticking, surface diffusion, adsorption and desorption phenomena as a function of gas pressure and temperature. Recently Arya et al. [33–34] suggested that for atomically smooth surfaces specular reflection may contribute to surface diffusion. While for pristine and uncoated CNT arrays and certain gas molecules this may be the case, it seems that the ALD coating process is simulated by diffusive reflection [27–28]. Here, we derive gas-transport formulae based on diffusive reflection in randomly distributed vertically aligned arrays of nanocylinders (nanotubes or nanowires). We would like to point out that our approach can also be used to derive expressions for diffusion accounting for specular reflections.

We present rigid derivations of the gas diffusion coefficient *D* inside such arrays, the time between collisions of molecules with nanocylinder walls, τ, the molecule surface impingement rate, *J*, and the Knudsen number of such a system, Kn. The derivations have been performed employing a random-walk model of gas molecules undergoing memoryless, diffusive reflection from nanocylinder walls assuming the molecular regime of gas transport. The example calculation of a diffusion coefficient is delivered for a system of titanium isopropoxide molecules diffusing between vertically aligned carbon nanotubes coated with titanium dioxide, which is especially relevant for applications in ALD.

## Foundations of the developed model

### Physical assumptions

We use the following assumptions for the gas-transport conditions within the model system of nanocylinders:

- The gas is dilute, meaning that intermolecular collisions can be neglected and within the space confined by nanocylinders the diffusion occurs in a molecular regime.
- The system of nanocylinders is of high aspect ratio, which from the point of view of diffusion means a large ratio of cylinder length to mean horizontal penetration depth.
- The system exhibits a continuous translational symmetry in transverse direction owing to the random cylinder distribution on the substrate surface, which allows us to reduce the three-dimensional diffusion problem to one dimension along the tube axis.
- Lambert’s cosine law is assumed for memoryless, diffusive re-emission (reflection) of gas molecules from the nanocylinder surface.

**Figure 1:**
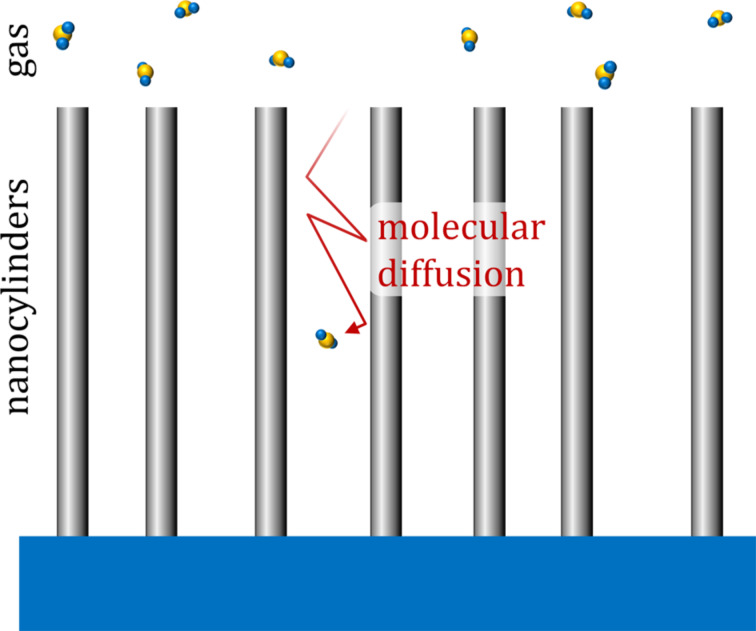
Schematic of an array of randomly distributed vertically aligned high-aspect-ratio nanocylinders. The gas is dilute, therefore collisions between molecules within the nanocylinder array can be neglected. Reflections of molecules from the walls are diffusive (cosine distributed).

For the sake of clarity, dimensions of a molecule are assumed much smaller than both the nanocylinder diameter and the flight distance of the molecules. The finite molecular dimension can be taken into account by extending the parameter of nanocylinder diameter *d* with the molecular diameter *d*_m_ resulting in 
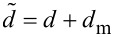
. The model system is schematically illustrated in Figure 1.

In the derivations we have employed a random-walk model of a molecule being diffusively reflected from the nanocylinder walls. Provided that there is no correlation between subsequent random-walk steps, according to the Einstein–Smoluchowski approach [35], the diffusion coefficient can be expressed as

[1]
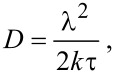


where λ^2^ is the mean square displacement, τ is the mean time of flight between two successive wall collisions and *k* is the number of dimensions in which the diffusion occurs. Attenuation of the molecule flux into the array by molecules hitting the top faces of the cylinders does not require consideration because the variable describing the diffusing species is solely the concentration of molecules within the volume of space unoccupied by obstacles.

### Definition of the system geometry

**Figure 2:**
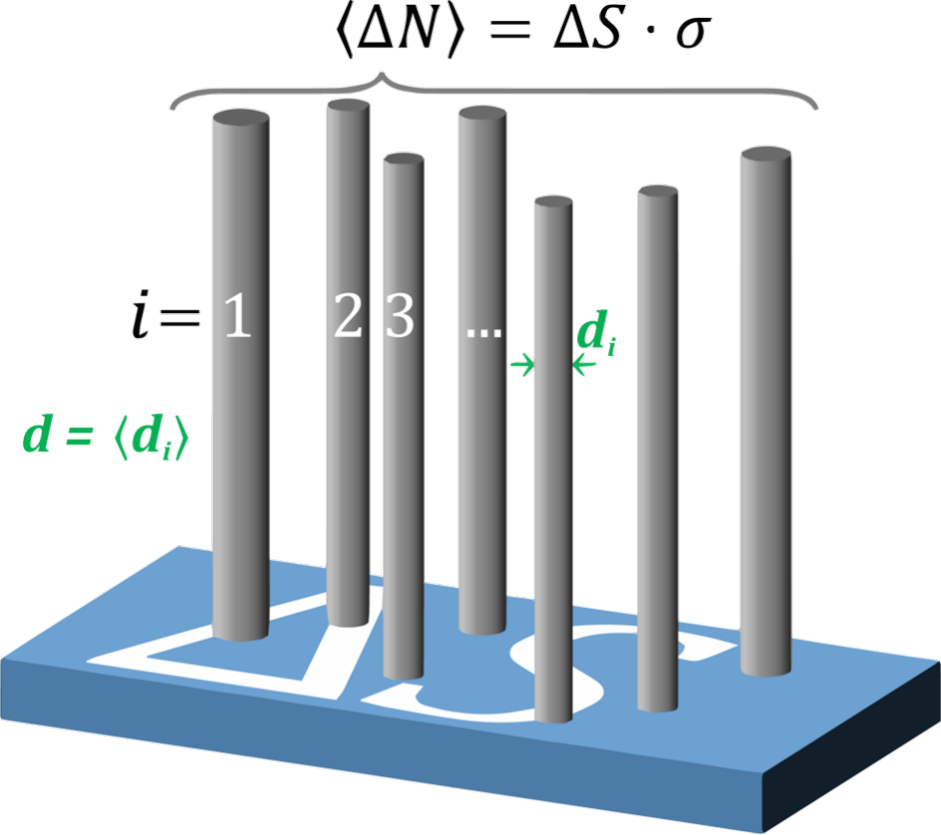
Schematic illustration of the geometrical model used to approximate an array of randomly distributed, vertically aligned, high-aspect-ratio nanocylinders of a mean diameter *d* and a surface number density σ. A flat substrate with an area Δ*S* is shown with a mean number of nanocylinders 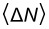 estimated as the product of Δ*S* and σ.

The geometry of a nanocylinder array is defined by the nanocylinder areal density, σ, and the mean nanocylinder diameter, *d*, as shown in Figure 2. The figure illustrates a mean number of nanocylinders 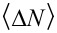 on a given substrate area Δ*S*, which can be estimated as the product of Δ*S* and σ.

## Results and Discussion

In this chapter we present derivations that emerge from the definitions and assumptions given above. In order to fully describe the kinematics of molecular diffusion, we deliver physical formulae describing i) the mean time of flight of a diffusant, τ, ii) the cylinder wall impingement rate, *J*, and iii) the diffusion coefficient, *D*. Additionally, we derive the Knudsen number Kn and define a gas-transport aspect ratio AR_array_, which allow us to assess whether the assumptions of molecular diffusion and high aspect ratio are justified in the given system and deliver an example calculation for a system that was proven to fulfil both assumptions. To initiate the random-walk approach, we describe the probability distributions of the transverse penetration distance, *x*, and a diffusive reflection angle θ, which will serve as the basis of the further derivations.

### Probability distribution of the transverse penetration distance

**Figure 3:**
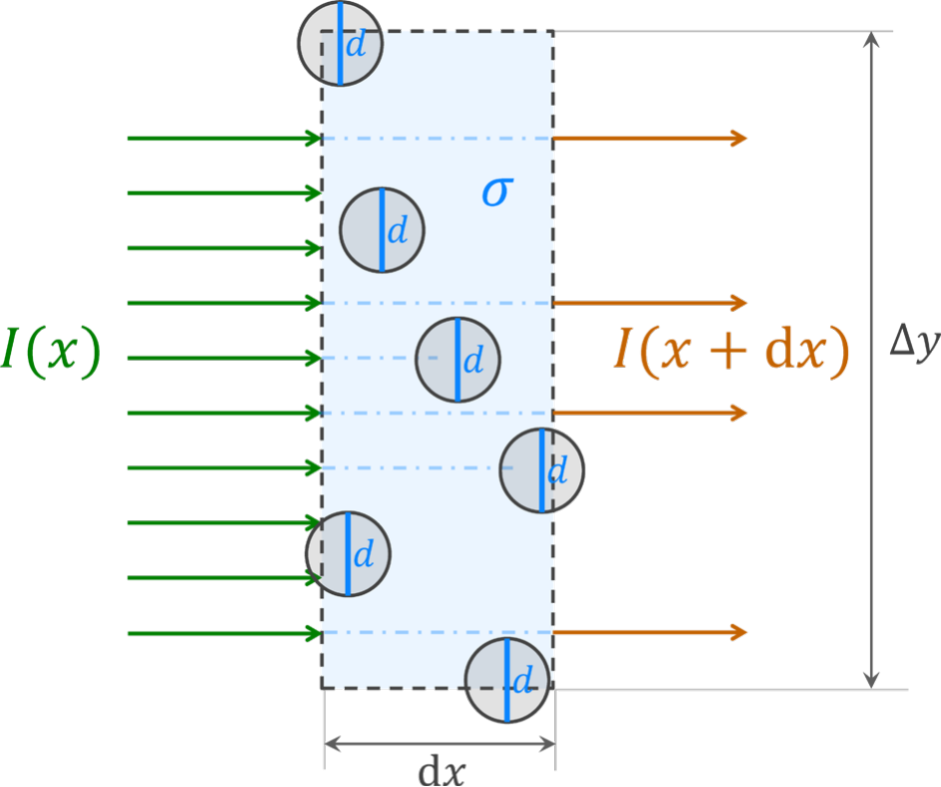
Top view schematic of the attenuation of a transverse flux *I* of molecules through the slice of a nanocylinder array of lateral dimensions Δ*x* × Δ*y*. For clarity, the diameters in the figure were kept constant instead of randomly distributed, which does not alter the result of the derivation.

Although the molecules travel in all three dimensions, the flight distance of a molecule is confined by the distance at which it hits another tube wall in transverse direction, as the tubes are assumed long and vertical. The distance travelled in the transverse direction by a molecule diffusing through the array of nanocylinders will be denoted as *x*. From a point of view of a lateral flux of molecules, *I*, the nanocylinders act as tall targets of width *d* and lateral areal density σ. Thus, the flux *I* is attenuated across the infinitesimal distance d*x* by a factor equal to a fraction of the transverse projection unoccupied by the cylinders, as shown in Figure 3.

The attenuation factor is equivalent to the probability of transmission through a slice of thickness d*x* denoted as *T*_d_*_x_*.

[2]
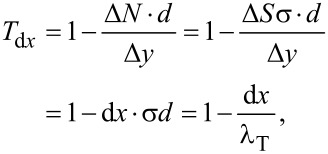


[3]
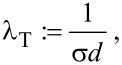


where the mean transverse penetration distance, λ_T_, is implicitly defined. Note that the above reasoning is sensitive solely to the mean value of *d* and not to the specific form of the diameter distribution. We can derive the transverse flux transmitted through a slice of the cross-section of a finite thickness *x*, obtaining a differential equation and its solution:

[4]
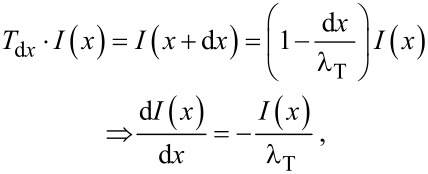


[5]
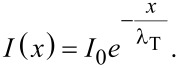


where *I*(*x* = 0) = *I*_0_ has been placed as an initial condition. Knowing that the penetration distance probability density function *p*(*x*) is proportional to the transmitted flux, we can obtain it by normalising the expression

[6]
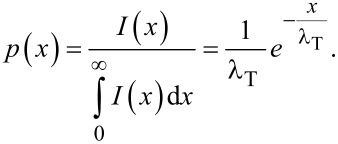


The probability density function is exponential, meaning that the described process is Poissonian. In a recent study the expression

[7]
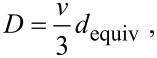


**Figure 4:**
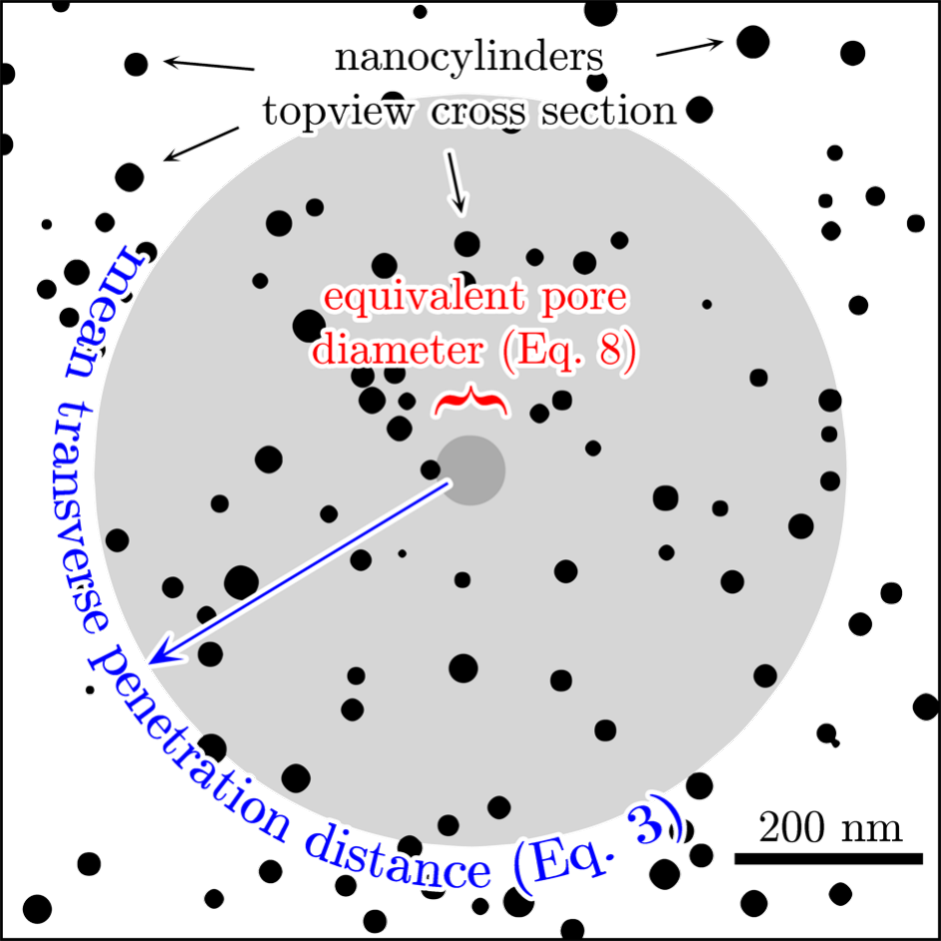
Illustration of the equivalent pore diameter (Equation 8) and the mean transverse penetration distance calculated using Equation 3. Diameters of nanocylinders are normally distributed with a mean value of 25 nm and a standard deviation of 6 nm. The nanocylinders are uniformly distributed on the substrate with an areal density of 10^10^ cm^−2^.

[8]
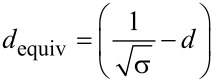


has been used as a pragmatic estimation of a lower limit of *D* in an array of vertically aligned carbon nanotubes [17], which constitutes an analogy to Knudsen diffusivity through cylindrical pores with the equivalent pore diameter designated as *d*_equiv_. The limit of this approach is illustrated with an example geometry in Figure 4 using a mean spacing of nanocylinders of 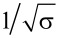 = 100 nm (σ = 10^10^ cm^−2^) and a mean cylinder diameter of 25 ± 6 nm as an example. It is evident, that the lower-limit guess strongly underestimates the mean transverse penetration distance. In the following we will develop the diffusion model towards a greater physical accuracy.

### Angular distributions of diffusive reflection

As mentioned in the assumptions above, the collisions of molecules with the nanocylinder walls are memoryless and diffusive, meaning that the solid angle of re-emission after a collision is governed by Lambert’s cosine law, independent of the angle of incidence [24,36]. The angle θ_0_ measured with respect to the normal, and the azimuthal angle φ_0_ have probability distribution functions given by

[9]
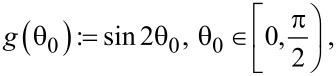


**Figure 5:**
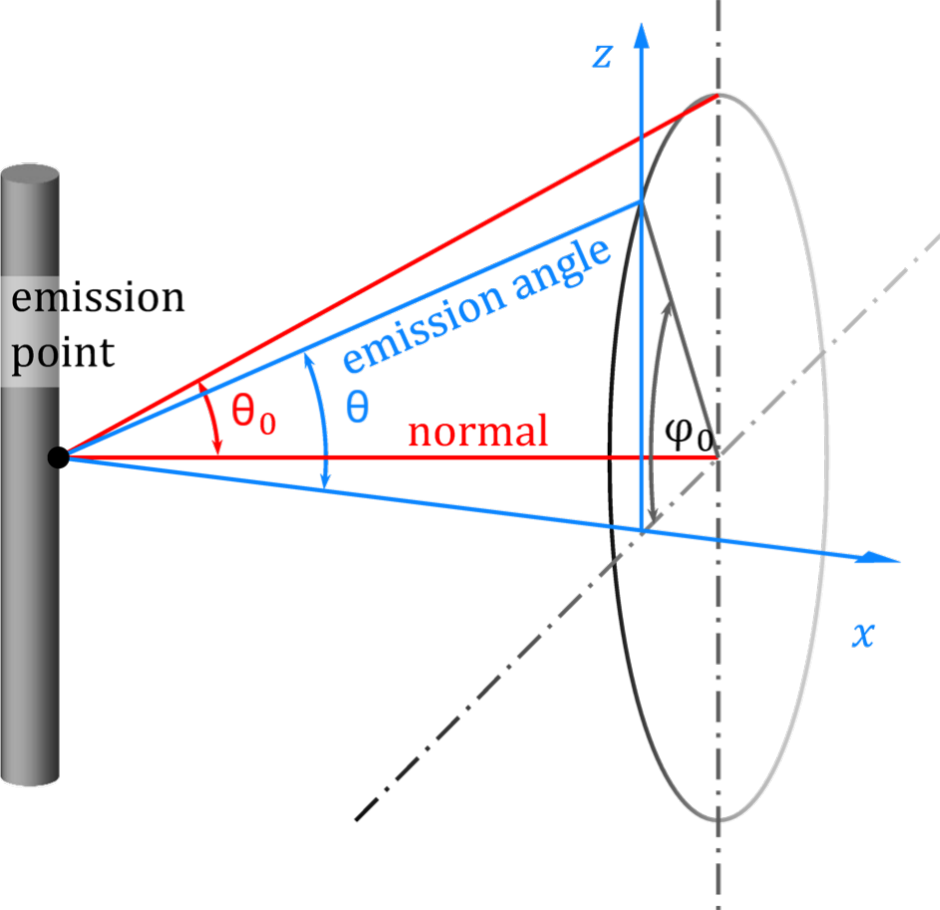
Schematic illustration of the angles defined above. The axis *x* is the direction of the transverse penetration distance with the probability density function given in Equation 6, whereas the axis *z* is the direction along the axis of the nanocylinders.

[10]
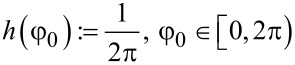


and they are independent of each other. The angles are visualized in Figure 5.

Considering the displacement of a molecule in the vertical direction along the axis of the nanocylinder, the only angle that is explicitly significant to the phenomenon is the angle in the vertical plane θ, which fulfils the condition

[11]
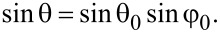


Introducing a set of temporary variables (*u*, *w* and *q*) and their probability distribution functions denoted as “pdf” with the according subscripts

[12]
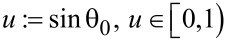


[13]
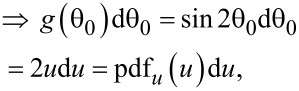


[14]
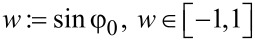


[15]
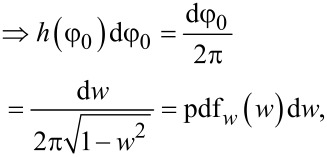


[16]
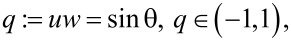


we can obtain the probability density function of *q* being a product of two independent random variables *u* and *w*:

[17]
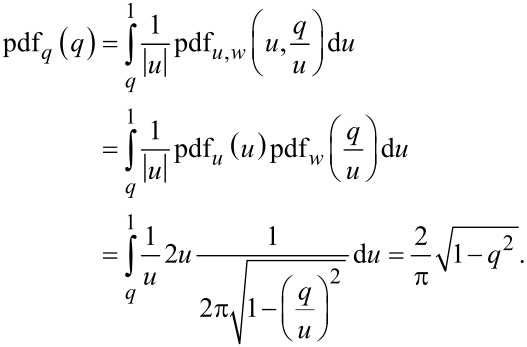


The integration limits are taken to be *q* and 1, while from Equations 12–16 it is evident that *u* is always greater than *q* and the maximum value of *u* is 1. Accordingly, we obtain

[18]
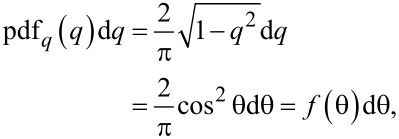


[19]
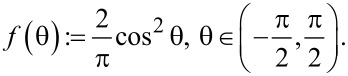


Thus, we have obtained the probability density function *f* of the vertical emission angle θ, which is necessary for the further derivations.

### Mean time of flight

Once the probability density functions are available, the time of flight can be derived. Knowing the transverse flight distance *x* and the angle in the vertical plane θ we calculate the flight distance:

[20]
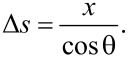


**Figure 6:**
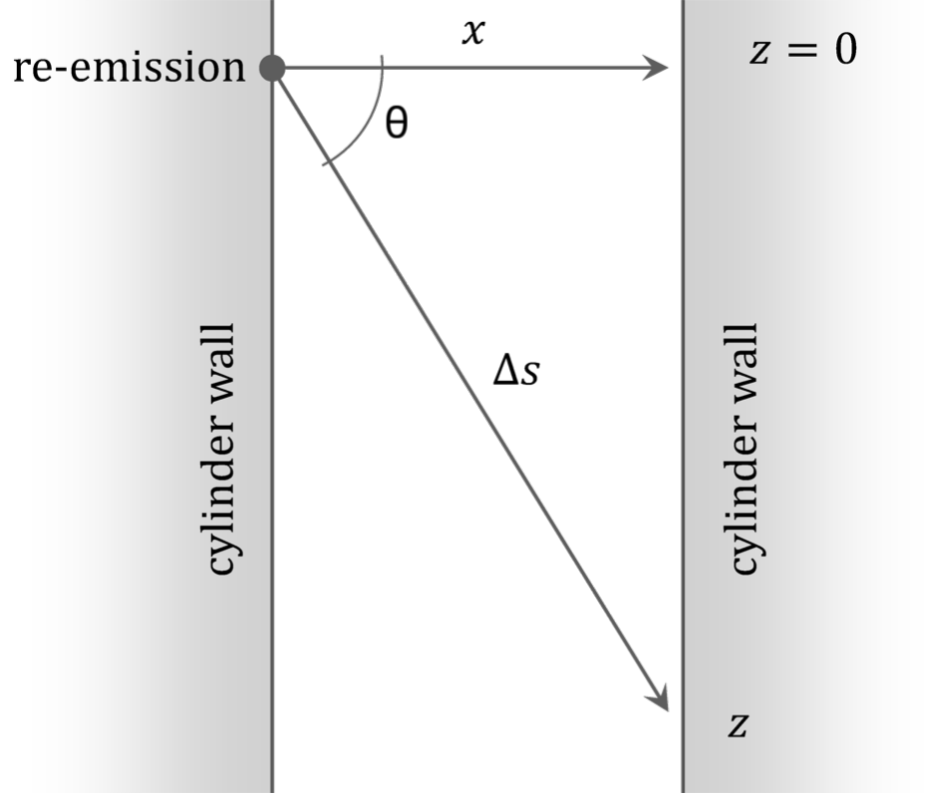
Schematic illustration of the flight geometry between two subsequent collisions of the molecule with a nanocylinder wall. The quantities in the figure are explained in the main text.

Equation 20 is geometrically implied in Figure 6. To obtain the mean value of Δ*s* a two-dimensional probability density function for both random variables *x* and θ is required. The variables are independent, therefore the distribution is the product of the two individual distributions:

[21]
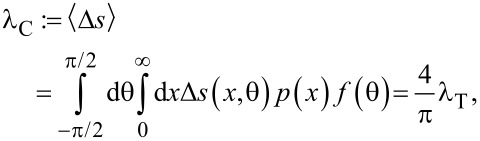


where the mean confined flight path λ_C_ has been defined. The mean time of flight is therefore equal to

[22]
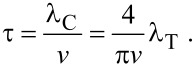


The velocity *v* can be taken as the mean absolute velocity from the Maxwell–Boltzmann distribution.

### Impingement rate

The mean time of flight τ can be translated to the more commonly used impingement rate *J* by definition from gas kinetic theory:

[23]
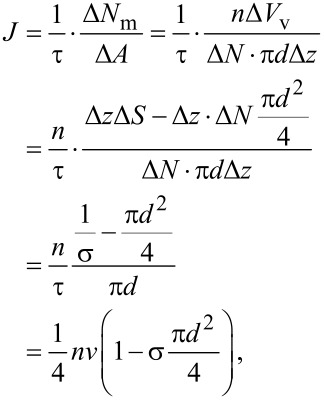


where Δ*N*_m_ refers to a number of molecules in an arbitrary volume within the nanocylinder array, Δ*A* is the area of nanocylinder walls in the given volume, *n* is the number volume density of molecules in the gas phase, whereas Δ*V*_v_ is the void volume between nanocylinders. The quantity σ·π*d*^2^/4 reflects the volume fraction occupied by the nanocylinders.

### Diffusion coefficient

At this point, the only quantity that is yet to be derived to fully express *D* is the mean square displacement in the longitudinal direction λ^2^. It can be obtained as the mean value of *z*^2^. We deduce the longitudinal displacement in one flight z from the geometry in Figure 6:

[24]
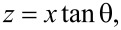


and derive λ^2^:

[25]
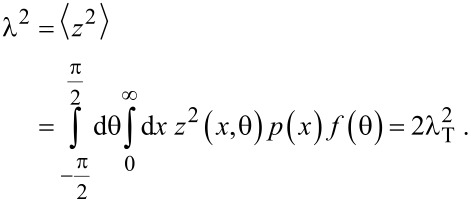


Using Equation 1, substituting τ obtained earlier and setting *k* = 1 as we consider only the longitudinal displacements, we can derive the equation for the diffusion coefficient *D* of gas molecules along an array of randomly distributed, vertically aligned, high-aspect-ratio cylinders:

[26]
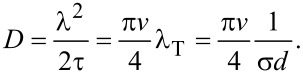


It is to be noted that the mean square transverse displacement is equal to the mean square longitudinal displacement

[27]
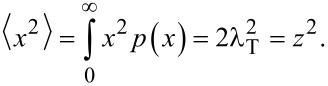


However, the diffusion coefficient in transverse direction *D*_T_ is different from the one in longitudinal direction. Laterally, the diffusion is two-dimensional (*k* = 2). Therefore it is equal to

[28]
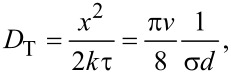


which is half the value of the longitudinal one, and, effectively, the diffusion is anisotropic. The anisotropic diffusion is modelled with the three-dimensional diffusion equation

[29]
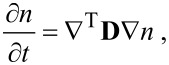


where *t* is time, 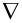 is the gradient column vector operator, the superscript “T” means transposition and **D** is the diffusion matrix. In our case, **D** takes the form

[30]
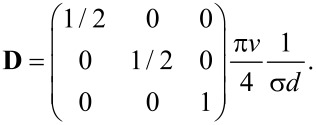


The off-diagonal terms in Equation 30 vanish because of the lacking correlation between the random motions of every pair of principal directions due to the random distribution of the vertical cylinders. The diffusion exhibits continuous translational and rotational symmetry around the direction of the cylinder axis. This would be violated by any correlation between the principal directions.

**Figure 7:**
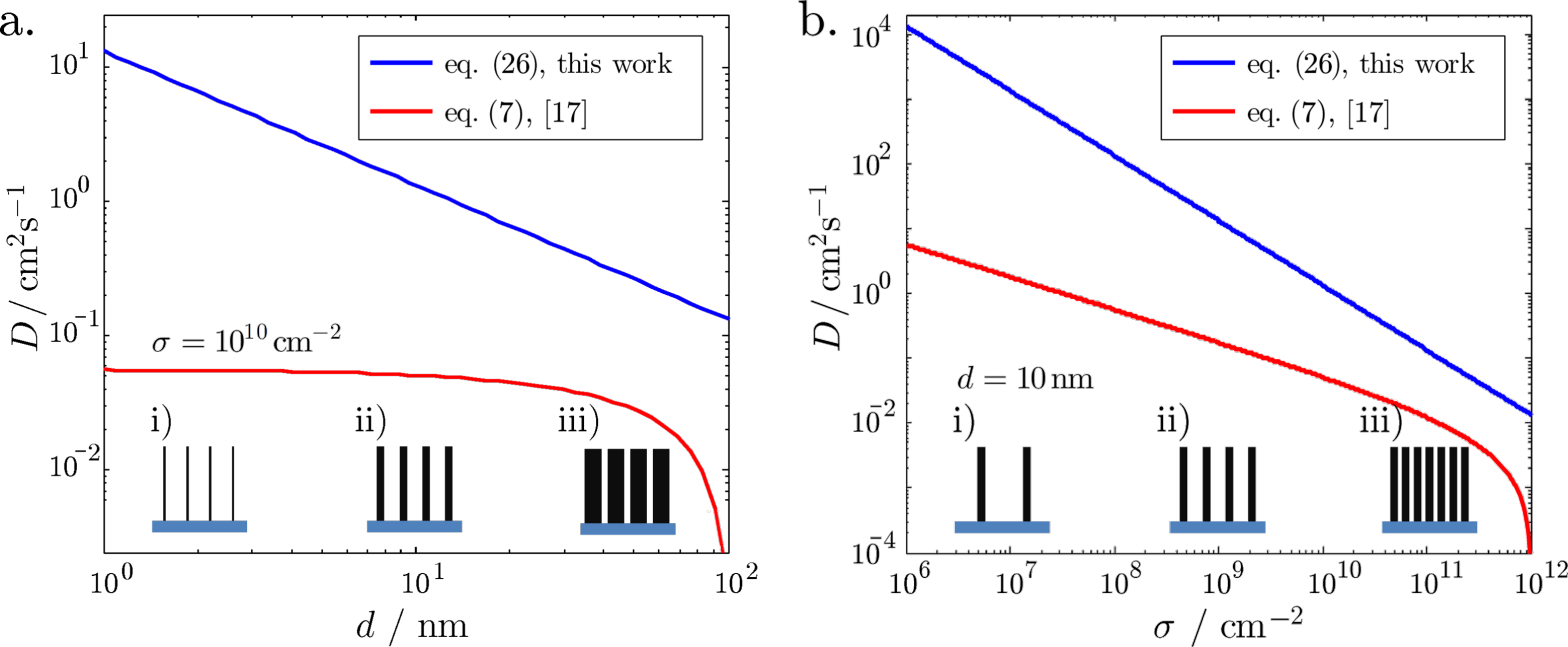
Comparison of the diffusion coefficient introduced in this work given by Equation 26 and the one from [17] given by Equation 7. The molecule velocity was taken as *v* = 171 m/s. a) Diffusion coefficient versus tube diameter *d* at a constant cylinder areal density σ = 10^10^ cm^−2^. b) Diffusion coefficient versus cylinder areal density σ at a constant mean diameter *d* = 10 nm. The insets show schematically the altered parameter of the structure on the horizontal axis (for the sake of clarity, the randomisation of diameters and positions are not shown).

In the next part we compare Equation 26 with the lower limit expression in Equation 7. The comparison of the two diffusion coefficients is shown in Figure 7. In the calculations, titanium tetraisopropoxide (TTIP), which is a widely used gas precursor in ALD of titanium oxide [37], was taken as an example. The molecular weight of the compound is *M* = 284.215 g/mol. The temperature was taken to be *T* = 100 °C. Taking the expression for the mean absolute value of velocity from a Maxwell–Boltzmann distribution of molecule velocities from the classical kinetic theory of gas, one obtains

[31]
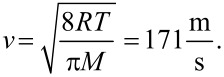


Numerical values of the diffusion coefficient using *v* = 171 m/s and Equation 26 are shown in Figure 7. In Figure 7a the areal density of nanocylinders σ = 10^10^ cm^−2^ was considered as an example of a high density of VACNTs grown by means of CVD [38] – the distance between the nanotubes then being estimated to be ≈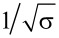 = 100 nm. The diffusion coefficients are compared within a range of nanocylinder diameters from 1 to 100 nm. Analogously, in Figure 7b, the coefficients are compared at a constant diameter of 10 nm, with varying areal density from 10^6^ to 10^12^ cm^−2^.

The two formulas result in qualitatively different estimations of *D*. The effective pore diameter approach from [17] (Equation 7) would strongly underestimate the diffusion coefficient within the entire range of the tested parameters.

### Knudsen number in an array of vertical randomly distributed nanocylinders

The molecular gas transport regime requires the mean flight path of molecules in a confined space λ_C_ to be much smaller than the mean free path in bulk gas λ_B_ at the same pressure and temperature. This condition implies that intermolecular collisions occur much less frequently than molecule–wall collisions, and consequently, their influence can be neglected. The ratio between λ_B_ and λ_C_ is called the Knudsen number

[32]
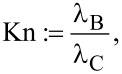


and its magnitude allows us to assess whether the gas transport occurs in the molecular regime. Conventionally, the regime is molecular, when Kn is greater than 10 [39–40]. The quantity λ_C_ is taken as a characteristic dimension of a porous system, i.e., the pore diameter, as it is an estimate of the mean confined flight path. Obviously, for arrays of nanocylinders it is not trivial to define a pore diameter. Instead, we derive an explicit formula for λ_C_ taking Equation 2 and Equation 21.

[33]
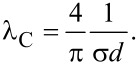


λ_B_ is known from classical molecular kinetics:

[34]
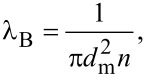


where *d*_m_ is the diameter of a molecule. This gives the Knudsen number

[35]
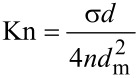


for an array of randomly distributed vertically aligned nanocylinders. The Knudsen number defined by Equation 4 can be used to verify whether a given system of nanocylinders with areal density σ and mean cylinder diameter *d* placed in a gas of an arbitrary concentration *n* and molecular diameter *d*_m_ constitutes a confined space for molecular gas transport, and, consequently, whether the kinetic parameters of the gas transport can be estimated using the equations provided in this work.

### The aspect ratio of gas transport in nanocylinder arrays

The applicability of our model is further confined by the aspect ratio of gas transport. For nanopores this ratio corresponds straightforwardly to the geometrical aspect ratio of pore length to pore diameter as the transverse molecule flight path is confined by the pore diameter. For nanocylinders arrays the transverse molecule flight path is given by λ_T_ (Equation 3) and the aspect ratio of gas transport AR_array_ becomes

[36]
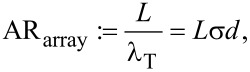


where *L* is the cylinder length. In analogy to Clausing [41] we can apply our model for AR_array_ > 10. For AR_array_ < 10 a transition to bulk gas diffusion takes place.

### Example calculation

We provide an example calculation of Kn and AR_array_ for the system of ALD of titania on VACNTs that appeared to be governed by molecular diffusion in the study of Yazdani and co-workers [17]. In this study the relevant parameters were the following: *d* = 10 nm, σ = 10^10^ cm^−2^, *L* = 100 μm, *n* = 5 × 10^15^ cm^−3^, *d*_m_ = 1 nm. Inserting these in Equation 35 and Equation 36, we obtain Kn = 50 and AR_array_ = 100, both being greater than 10 which lies within the applicability range of our model.

## Conclusion

The diffusive transport of dilute gas in an array of vertically aligned system of randomly distributed high-aspect-ratio cylinders of random diameters has been discussed. New formulae have been derived for (i) the mean flight path in space confined by such an array of cylinders, (ii) the mean time of flight, (iii) the impingement rate, (iv) the Knudsen number, and ultimately (v) the diffusion coefficient for such a system using a probabilistic random walk model.

The formulae obtained are of a great assistance in understanding quantitatively the process of gas transport in arrays of vertically aligned cylinders, such as nanotubes or nanowires of various materials. This is crucial for the optimisation of gas-based surface-functionalisation processes of such arrays as well as of the gas sensing performance of such systems, CVD growth of carbon nanotubes or any other fields where gas transport between nanowires or nanotubes is considered.

## References

[1] Chan C K, Peng H, Liu G, McIlwrath K, Zhang X F, Huggins R A, Cui Y (2008). Nat Nanotechnol.

[2] Song T, Xia J, Lee J-H, Lee D H, Kwon M-S, Choi J-M, Wu J, Doo S K, Chang H, Park W I (2010). Nano Lett.

[3] Fan S, Chapline M G, Franklin N R, Tombler T W, Cassell A M, Dai H (1999). Science.

[4] Kijima T, Yoshimura T, Uota M, Ikeda T, Fujikawa D, Mouri S, Uoyama S (2003). Angew Chem.

[5] Kasuga T, Hiramatsu M, Hoson A, Sekino T, Niihara K (1999). Adv Mater.

[6] Xiao Z L, Han C Y, Welp U, Wang H H, Kwok W K, Willing G A, Hiller J M, Cook R E, Miller D J, Crabtree G W (2002). Nano Lett.

[7] Park H-G, Kim S-K, Song K-D, Kempa T J, Lieber C M (2016). Multishell nanowires for next-generation photovoltaics. Progress in Electromagnetic Research Symposium (PIERS).

[8] Wang C-C, Hsueh Y-C, Su C-Y, Kei C-C, Perng T-P (2015). Nanotechnology.

[9] Ye T, Jun L, Kun L, Hu W, Ping C, Ya-Hui D, Zheng C, Yun-Fei L, Hao-Ran W, Yu D (2017). Org Electron.

[10] Lu X, Yu M, Wang G, Zhai T, Xie S, Ling Y, Tong Y, Li Y (2013). Adv Mater.

[11] Lau K K S, Bico J, Teo K B K, Chhowalla M, Amaratunga G A J, Milne W I, McKinley G H, Gleason K K (2003). Nano Lett.

[12] Marichy C, Bechelany M, Pinna N (2012). Adv Mater.

[13] Qin Y, Lee S-M, Pan A, Gösele U, Knez M (2008). Nano Lett.

[14] Zhang Y, Guerra-Nuñez C, Li M, Michler J, Park H G, Rossell M D, Erni R, Utke I (2016). Chem Mater.

[15] Li J, Lu Y, Ye Q, Cinke M, Han J, Meyyappan M (2003). Nano Lett.

[16] Chen J, Xu L, Li W, Gou X (2005). Adv Mater.

[17] Yazdani N, Chawla V, Edwards E, Wood V, Park H G, Utke I (2014). Beilstein J Nanotechnol.

[18] Zhong G, Iwasaki T, Robertson J, Kawarada H (2007). J Phys Chem B.

[19] Xiang R, Yang Z, Zhang Q, Luo G, Qian W, Wei F, Kadowaki M, Einarsson E, Maruyama S (2008). J Phys Chem C.

[20] Poodt P, Cameron D C, Dickey E, George S M, Kuznetsov V, Parsons G N, Roozeboom F, Sundaram G, Vermeer A (2012). J Vac Sci Technol, A.

[21] Louchev O A, Laude T, Sato Y, Kanda H (2003). J Chem Phys.

[22] Zhu L, Hess D W, Wong C-P (2006). J Phys Chem B.

[23] Knudsen M (1909). Ann Phys.

[24] Knudsen M (1916). Ann Phys.

[25] Roy S, Raju R, Chuang H F, Cruden B A, Meyyappan M (2003). J Appl Phys.

[26] Roy S, Cooper S M, Meyyappan M, Cruden B A (2005). J Membr Sci.

[27] Gordon R G, Hausmann D, Kim E, Shepard J (2003). Chem Vap Deposition.

[28] Detavernier C, Dendooven J, Pulinthanathu Sree S, Ludwig K F, Martens J A (2011). Chem Soc Rev.

[29] Elam J W, Routkevitch D, Mardilovich P P, George S M (2003). Chem Mater.

[30] Elam J W, Pinna N, Knez M (2011). Coatings on High Aspect Ratio Structures. Atomic Layer Deposition of Nanostructured Materials.

[31] Panda S K, Shin H, Pinna N, Knez M (2011). Step Coverage in ALD. Atomic Layer Deposition of Nanostructured Materials.

[32] Yanguas-Gil A, Yang Y, Kumar N, Abelson J R (2009). J Vac Sci Technol, A.

[33] Arya G, Chang H-C, Maginn E J (2003). Phys Rev Lett.

[34] Arya G, Chang H-C, Maginn E J (2003). Mol Simul.

[35] Einstein A (1956). Investigations on the Theory of the Brownian Movement.

[36] Greenwood J (2002). Vacuum.

[37] Aarik J, Aidla A, Uustare T, Ritala M, Leskelä M (2000). Appl Surf Sci.

[38] Tong T, Zhao Y, Delzeit L, Kashani A, Meyyappan M, Majumdar A (2007). IEEE Trans Compon Packag Technol.

[39] Lafferty J M (1998). Foundations of vacuum science and technology.

[40] Laurendeau N M (2005). Statistical thermodynamics: fundamentals and applications.

[41] Clausing P (1932). Ann Phys.

